# Between languages and scalpels: a woman's journey in a foreign neurosurgery residency

**DOI:** 10.3389/fsurg.2026.1848082

**Published:** 2026-06-10

**Authors:** Stephany Morales-Viquez

**Affiliations:** Department of Neurosurgery, Hadassah Hebrew University Medical Center, Jerusalem, Israel

**Keywords:** language barriers, mentorship, multilingual healthcare, neurosurgery, residency

## Introduction

Starting residency abroad did not simply mean learning a new hospital system. This meant relearning how to listen, ask, report, and participate under pressure, while still building the technical and cognitive foundations of neurosurgical training. In that context, language was not peripheral to training; rather, it was one of the conditions under which training was conducted.

Hebrew quickly became central to daily work. It was the language of ward rounds, operating room exchanges, bedside reassessment, urgent decision-making, and much of the informal teaching that shaped early residency. The challenge was not only grammar. Spoken clinical Hebrew is fast, compressed, context-dependent, and layered with abbreviations, idioms, and service-specific shorthand terms. In a high-acuity environment, delayed comprehension can affect not only confidence but also timing, participation, and perceived credibility. More broadly, language barriers in healthcare are associated with impaired communication, lower patient understanding, reduced trust, and greater strain on clinical teams (1–3).

I chose to train in Israel, specifically at Hadassah, because of the opportunity to learn in a high-volume academic center with broad neurosurgical pathology, strong operative mentorship, and a culturally diverse patient population. I underestimated how much of the first year would involve acquiring functional fluency while simultaneously learning to think and act as a neurosurgical resident. Published accounts from international physicians working in other health systems suggest that this combination of language learning, hidden curricular expectations, and gradual professional integration is more common than exceptional (4–14).

My transition to direct responsibility was also staged. I had approximately 6 months before I began taking calls and assuming direct overnight responsibilities, which gave me time to learn the hospital workflow, electronic systems, and functional Hebrew. In my local program, there was no separate formal Hebrew examination; instead, readiness was assessed through daily supervised interactions—conversing with patients, attendings, nurses, secretaries, and ancillary staff, and navigating a hospital environment, where both spoken and written communication was predominantly in Hebrew. For patients who spoke Arabic or Russian, official interpreter services were available, and the multilingual character of the hospital often meant that colleagues or staff could help bridge routine bedside communication when necessary. These local details are offered as context rather than as universal models. Across health systems, some programs rely more heavily on formal examinations, while others rely on supervised clinical performance. What matters analytically is that the linguistic threshold for safe participation must be defined, whether through formal testing, graded responsibility, longitudinal supervision, or a combination of these approaches.

### Why this experience matters

Personal narratives are most useful in academic writing when they illuminate broader professional questions. The question here is not whether adaptation abroad is difficult; that is already clear, and it is certainly not unique to one person, specialty, or country. This does not suggest that international neurosurgical trainees need a separate educational framework. Rather, it uses one local experience to examine a broader issue: how linguistic adaptation is lived in the early, high-acuity environment of residency, how it shapes access to learning, and what forms of support appear both realistic and useful.

This experience is not unique, nor is it confined to neurosurgery. Similar tensions have been described among newly employed international physicians in Norway, family medicine residents in Canada, international medical graduates in Australia and New Zealand, and fellows transitioning into new host systems, where communication, hierarchy, and hidden curricular expectations shaped integration (4–14). Across these studies, international trainees repeatedly described rapid clinical speech, uncertainty around unwritten norms, concerns that delayed expression may be misread as limited competence, and the importance of mentorship and social support. Even outside neurosurgery, residency programs have recognized language acquisition as part of clinical training, as illustrated by a second-language curriculum developed within anesthesiology residency (10). Therefore, the value of this perspective is not in claiming singularity, but in showing how a broader international training problem acquires a particular texture inside neurosurgery.

This perspective is also narrower than a general statement about the diversity policy. The practical concern is whether a trainee entering a multilingual, high-acuity surgical service can participate, learn, and care for patients safely. Gender is addressed here as one modifier of that experience, not as the sole explanatory frame. Therefore, this study examines five linked processes: learning in a multilingual environment, communicating with neurologically vulnerable patients and families, integrating into a new clinical culture, negotiating the interaction between language and gendered expectations, and balancing trainee responsibility with program responsibility. The literature on professional identity formation and support for international medical graduates suggests that these processes are socially mediated rather than purely individual (11–17).

### Learning in a multilingual environment

At the outset of my residency, I expected Hebrew to be the principal linguistic barrier. In practice, the environment was more complex. English often functioned as a partial bridge with colleagues, while some patients communicated primarily in Arabic or Russian. I did not aim for full proficiency in all the languages represented in the hospital. Instead, I learned a limited set of high-yield phrases for greeting, symptom assessment, orientation, focused neurological examination, and postoperative reassessment, and I relied on bilingual colleagues, official interpreters, and other appropriate communication support when more detailed discussions were required (1–3, 11, 17).

The cognitive load of multilingual processing was found to be substantial. During rounds or in the operating room, I often received information in one language, translated it internally into another language, and formulated a response in a third language. Early on, that delay could make me appear to be slower than I was. Repeated exposure to routine consultations and standard postoperative questions gradually reduced this friction. A practical lesson emerged: in residency, fluency is not acquired before participation; much of it is consolidated through supervised participation.

Much of this learning was informal. It was the other Latin American co-residents who first taught me to effectively communicate with a patient in Hebrew to obtain a history and perform a basic examination. This was important because language courses start with ordinary conversation rather than focusing on specific issues like focal weakness, sensory loss, visual fields, neglect, aphasia, postoperative warning signs, or the concise language used in urgent reassessment. Much of my clinical vocabulary has come from repeated use in the ward rather than from formal study alone. Comparable workplace-based learning has been reported by international medical graduates in other systems, who similarly described peers, supervisors, and repeated clinical exposure as major sources for practical language acquisition (4–14).

The attendings also contributed in practical ways. During rounds or in the operating room, they sometimes taught me a new word, explained the root of a term, or connected a phrase to a linguistic pattern that made it easier to remember. These were small interventions, but they made the language feel learnable and signaled that language learning did not have to remain a private struggle after-hours. This kind of everyday supervisory support is consistent with broader evidence that the integration of international trainees is facilitated by local coaching, structured feedback, and inclusion in routine clinical work (11–17).

### Patients, families, and neurological examination

My lack of fluency not only affected how I spoke, but it also changed how I examined patients. In neurosurgery, this distinction matters because language is often part of the disease itself. Patients may be aphasic, dysarthric, cognitively slowed, confused, or too ill to provide a coherent history of their disease. Therefore, communication is part of the neurological assessment and not a separate task.

When I encountered patients who spoke only Arabic or Russian, official interpreter services were available, and the multilingual composition of the staff often provided an additional bridge for routine, bedside communication. However, bedside assessment often requires adaptation in real time. For an initial focused evaluation, I sometimes relied on family members to help relay brief questions and answers, or I asked nurses, technicians, or bilingual colleagues to help bridge the exchange until more formal support was established. Even when a patient spoke Hebrew, there were moments when my phrasing was imprecise, and the relatives helped interpret what I was trying to ask. These encounters made me more deliberate in choosing simple, functional language, and more disciplined in pairing verbal questions with observation and examination.

Patients reacted in varied but revealing ways to answer the questions. Some immediately recognized that I was still learning Hebrew and met me halfway by simplifying their speech, repeating themselves more slowly, or shifting parts of the conversation into English. At times, we moved naturally between both languages in the same encounter. Some sought confirmation from a family member first, which reminded me that fluency influences not only the transfer of information but also the perception of authority. However, in most cases, the response was not rejection but collaboration. Patients and families often showed patience when they understood that the examination was careful, serious, and purposeful. This observation aligns with the broader literature showing that language discordance influences trust, comprehension, and perceived quality of the clinical encounter (2, 3, 6, 7).

These experiences also shaped my approach to difficult conversations. When I needed to explain a patient's condition, discuss the prognosis, or summarize neurological changes, I often relied on co-residents or nurses to help ensure that the explanation was accurate and understandable. When more complex communication was anticipated, formal interpretation was the safer option. In urgent moments, however, clinical communication remained collaborative and depended on the wider care team.

Because I could not always communicate quickly, I became more observant of facial expressions, hesitations, breathing patterns, asymmetries, behavioral changes, and the difference between spoken words and clinically evidence. Observation is not a substitute for language; however, in neurosurgery, it is an essential complement. Paradoxically, limited fluency made me a more attentive bedside observer.

### Gender as a modifier of the experience

Gender is addressed here as a modifier of the experience rather than as its primary explanation. I do not argue that multilingual training is uniquely difficult for women or that women and men learn languages differently. In a historically male-dominated specialty, linguistic hesitation may at times be interpreted through unequal expectations of confidence, decisiveness, and authority. In a narrower sense, gender shaped how this experience was lived (18–22).

My experience was defined by more than just gender, and not every difficulty I faced was a result of it. However, being a woman in neurosurgery influenced how I experienced visibility, belonging, and credibility while also being new to the language and local system. A pause to find the right word can be read as an ordinary adjustment in one trainee and as uncertainty in another. In the initial stages of training, language can blur the distinction between not knowing something and not yet being able to express what one knows quickly. For women in a surgical environment, this blurring can carry additional weight because confidence and authority are often judged through speech, brevity, and decisiveness. Broader surgical literature has also described how women's authority may be filtered through gendered expectations about confidence, leadership, and belonging (22).

Gender also shapes the importance of mentorship and representation. Seeing women in neurosurgery and receiving support from colleagues who understood the demands of the specialty made the sense of belonging feel more attainable. The literature on women in neurosurgery has emphasized persistent concerns regarding mentorship, visibility, retention, and access to opportunities (18–21). My experience aligns with the literature in a specific way: while gender did not replace language as the primary challenge, it influenced how that challenge was interpreted, how comfortable I felt speaking before I felt fluent, and how important it was to be corrected without being diminished.

### Integration into a new team and clinical culture

Vocabulary alone was not sufficient. Adaptation also required understanding how communication functioned within the local clinical culture: knowing when to present briefly and directly, when to seek immediate clarification, how much contextual detail was expected in a patient presentation, and how much teaching occurred during rapid clinical work. These norms are often invisible to those trained within the system, but they are central to integration for international trainees (5, 8, 9, 11–15, 18).

Much of this learning occurred through repeated small corrections rather than through formal orientation. A nurse would rephrase the sentence to make it sound natural. A colleague explained why a familiar word had a different practical meaning in the ward. During call shifts, staff members sometimes taught me colloquial expressions that made routine interactions easier. Although each moment was minor on its own, together they transformed the hospital into a language-learning environment embedded within clinical work.

In this context, mentorship was valuable not as an abstract ideal but as a set of specific behaviors. Senior surgeons corrected my presentations, clarified operative reasoning when I missed part of a discussion, and expected visible improvement while still allowing room for growth. Co-residents normalized the difficulty of the first few months and encouraged persistence. Nurses, technicians, and ward staff often assisted with phrasing, logistics, and orientation. The existing literature on international trainees and women in neurosurgery supports the importance of social support, structured transition, and mentorship during early adaptation (8, 11–14, 17–21).

This experience also changed my understanding of confidence in residency. At the beginning of the training, confidence can appear to be speed: immediate answers, rapid patient presentations, or effortless participation in discussions. In a foreign-language environment, however, confidence may first take a quieter form: asking again when something is unclear, speaking despite an accent, accepting correction without withdrawing, and continuing to participate before speech feels fully natural. For an international resident, professional identity develops through repeated negotiations between competence and expression. The literature on professional identity formation supports the idea that identity develops through participation, reflection, and social recognition (15, 16). In this sense, fluency and belonging do not develop sequentially, but they develop together.

### Balancing trainee responsibility and program responsibility

A balanced account must also acknowledge the agency. Choosing to enter residency in a new language is usually a choice rather than a situation forced upon someone without their consent, and those who pursue that path carry significant responsibility. They should arrive well-prepared, accept that the initial months will require accelerated language learning, and understand that patient care in high-acuity settings cannot depend indefinitely on others’ improvisation. This does not weaken the argument; rather, it clarifies it.

Nonetheless, programs play an important role. Their responsibility is not to eliminate the difficulty of adaptation or to create unlimited customized services for every trainee. Many hospitals operate under real financial and logistical constraints, and extensive resident-specific language infrastructure may be neither feasible nor necessary for them. The more relevant question is whether a program clearly defines expectations, monitors whether communication is safe, and reduces avoidable barriers to learning and patient care.

In practice, the most valuable support is often modest. A staged transition before independent calls, explicit feedback on presentations and bedside communication, ready identification of who can help in urgent multilingual situations, and a low threshold for professional interpretation in high-stakes conversations are all more feasible than building entirely new service lines. The literature on international trainee transition supports a structured yet realistic approach. This includes targeted induction, supervised participation, buddying, and ongoing feedback, which are more effective than assuming that adaptation will occur invisibly and without institutional attention (10–14, 17).

This balance also addresses a common false choice in the debate. The alternatives do not consist of complete institutional responsibility on the one hand and only complete individual responsibility on the other. Safe integration depends on both factors. Trainees must prepare seriously and continue learning actively, and programs must make their expectations explicit and judge language competence in relation to actual clinical tasks.

### Implications for training programs

Therefore, the argument in this study is narrower than the demand for expansive new services. Language barriers are not unique to neurosurgery, and not all international trainees require the same support. The claim is that language can meaningfully shape learning, confidence, bedside authority, and participation in early residency, and that training programs can reduce unnecessary barriers through feasible practices.

Hospitals that train multilingual residents should consider a more explicit structure for integrating linguistics. Instead of relying on a general language study to address specific needs, early orientation could include common phrases for neurological examinations, postoperative reassessments, emergency escalations, and family communication. New international residents could be paired with senior residents during their initial months, not only for clinical logistics but also for linguistic modeling: learning how to present concisely, ask focused questions, and clearly explain uncertainty at the bedside (10–14, 17).

Programs should also establish guidelines for when professional interpretation is required, particularly for consent processes, major prognostic discussions, and other important conversations. At the same time, they should recognize that bilingual staff may still help bridge urgent basic communication during immediate clinical assessments (1–3). Departments can normalize brief real-time corrections during rounds and in the operating room so that language learning becomes part of supervised practice rather than a hidden struggle. For services with recurrent multilingual encounters, short, specialty-specific language guides or simulation scenarios may be more realistic than resource-intensive formal curricula.

A culture of belonging also depends on how teams interpret this hesitation. When a trainee pauses to find the right word, that pause should not automatically be read as a lack of knowledge or poor judgment. Supervisors who distinguish remediable language difficulties from true clinical deficiencies help protect both learning and patient care. For international women trainees, this distinction may be especially important because language hesitation can be layered onto preexisting assumptions about authority in surgical spaces (18–22).

For international trainees, progress is often first visible in small but meaningful milestones, such as understanding a complete case discussion, giving a concise presentation without translation pauses, recognizing humor during rounds, or following operative teaching in real time. Although these achievements seem modest, they hold significant professional value because they mark the point at which a foreign training environment begins to feel merely a test of endurance and more like a place where one can genuinely learn. At that point, the discussion moves beyond memoir and toward educational design: the issue is no longer only how an individual adapts but how an institution makes safe learning more possible.

## Conclusion

Language should be understood as a structural component of surgical training for international residents rather than as a private obstacle to be overcome individually. In my experience, adaptation is possible through repetition, inclusion, and mentorship embedded in daily work. It was shaped not only by the effort to learn Hebrew but also by the responses of patients, families, nurses, co-residents, and attending surgeons who made communication a shared clinical task rather than an individual weakness to conceal.

At the same time, a balanced account must acknowledge that entering a new-language residency is a voluntary professional choice that carries the responsibilities of preparation, humility, and sustained effort. Programs are not required to eliminate this challenge, but they should clearly define linguistic expectations, protect high-stakes communication, and adopt feasible practices that make early participation safer and more educationally productive.

For a woman training in neurosurgery abroad, language and gender did not function as separate themes; they intersected in everyday negotiations of authority, belonging, and professional voice. The value of this perspective lies precisely in its focus—not on claiming novelty or calling for a separate training structure for foreign physicians, but on arguing that recurrent difficulties deserve explicit, proportionate, and specialty-aware attention if high-acuity training programs aim to support trainees and ensure patient safety.
